# Clinical and Dermoscopic Features of an Extradigital Glomus Tumor of the Back

**DOI:** 10.5826/dpc.1004a77

**Published:** 2020-10-26

**Authors:** Maha Lahouel, Ines Lahouel, Yosra Soua, Mouna Ben Hamouda, Manel Njima, Monia Youssef, Jameleddine Zili

**Affiliations:** 1 Department of Dermatology, CHU Fattouma Bourguiba, Monastir, Tunisia; 2 Department of Pathology, CHU Fattouma Bourguiba, Monastir, Tunisia

**Keywords:** glomus tumor, back, extradigital, dermoscopy

## Introduction

Glomus tumors (GT) are uncommon, usually benign, slow-growing, painful lesions [1]. They are frequently solitary and located on the extremities, mostly in the subungual areas of the digits [1]. Extradigital GT are rare, and little is known of their clinical and dermoscopic features, which explains frequent diagnostic delays. We present a case of an extradigital solitary glomus tumor located on the back, and we describe its dermoscopic features in such an exceptional location.

## Case Presentation

**Figure 1:**
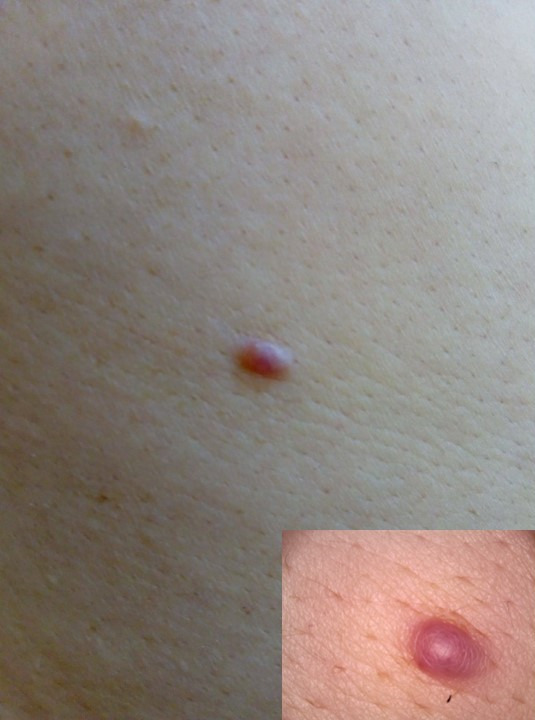
Clinical aspect: A purple well-defined papule on the back.

**Figure 2:**
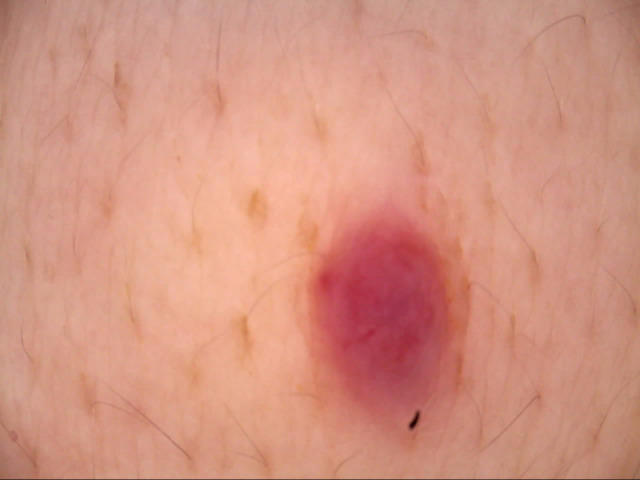
Polarized, non-contact dermoscopy showed a structureless purple area.

**Figure 3:**
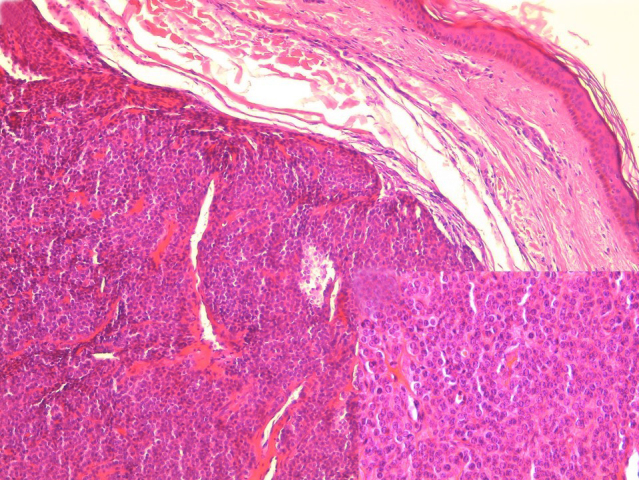
(A) Dermal solid sheet of blue cells with a thin fibrous capsule (H&E, ×40). (B) The cells are uniform with indistinct borders, eosinophilic cytoplasm, round nuclei and bland chromatin (H&E, ×400).

A 64-year-old woman presented with a 1-year history of a slow-growing painful nodular lesion on the back. The pain worsened with movement or mild touch, including simple contact with clothing. Physical examination revealed a well-defined, smooth, purple papule 8 mm in diameter (Figure 1). Polarized, non-contact dermoscopy showed a structureless purple area (Figure 2). An excisional biopsy was performed. Histopathology confirmed the diagnosis, showing a tumor enveloped by a fibrous capsule with vascular spaces surrounded by glomus cells (Figure 3). The excision was followed by immediate disappearance of the pain without recurrence to this day.

Glomus tumors are uncommon neoplasms of the glomus body [1]. GT account for less than 2% of all soft tissue tumors [1]. They usually present as a blue-to-pink soft nodule associated with a classic triad of symptoms: pain, pinpoint tenderness, and cold sensitivity [2]. These tumors tend to be small (<2 cm), frequently solitary, and located on the extremities, mostly in the subungual areas of the digits [2]. However, they can be found anywhere on the body. Extradigital cases reported in the literature correspond to 26.7% of all GT [2]. Extradigital localizations are (in decreasing order of frequency): the upper extremities, lower extremities, trunk, and face [1]. Nevertheless, to the best of our knowledge, the occurrence on the back, as the case of our patient, is extremely rare.

Extradigital GT differ in some clinical features from digital GT. Patients with extradigital GT are older than those with digital GT [1]. While there is an equal incidence of all GT in both men and women, extradigital GT are found more commonly in men [2]. Symptoms such as pain and hypersensitivity to cold are less frequently observed in patients with extradigital GT, leading to misdiagnosis [1]. The most common misdiagnoses were hemangioma, neuroma, and neurofibroma [1].

Dermoscopic features of extradigital GT have been documented in 5 previous cases [2]. Dermoscopic presentation revealed homogeneous, structureless, patternless lesions in all cases; a multicolored background, including a central purple area and a peripheral whitish homogeneous area in 1 case; yellow-to-white background in 3 cases; and multiple peripheral telangiectasias on the surface (2 cases). A structureless purple area, as seen in this observation, was described in 1 case. The central purplish structureless area correlates to enlarged vessels within the neuromyoarterial glomus body, while the surrounding white homogeneous area most likely corresponds to its fibrous capsule.

## Conclusions

Extradigital locations of GT may represent a diagnostic challenge. While not specific, dermoscopy provides additional clues to complement the differential diagnosis of GT in less common locations. However, confirmation requires histopathological study after surgical biopsy.
